# Visceral subpleural hematoma of the left diaphragmatic surface following left upper division segmentectomy

**DOI:** 10.1186/s13019-017-0657-6

**Published:** 2017-10-30

**Authors:** Yasushi Mizukami, Nobuhito Ueda, Hirofumi Adachi, Jun Arikura

**Affiliations:** Department of Thoracic Surgery, National Hospital Organization, Hokkaido Cancer Center, 2-3-54 Kikusui 4-jo, Shiroishi-ku, Sapporo-shi, Hokkaido 003-0804 Japan

**Keywords:** Visceral subpleural hematoma, Lung resection, Vats, Segmentectomy

## Abstract

**Background:**

Pulmonary visceral subpleural hematoma is rare. We report visceral subpleural hematoma of the left diaphragmatic surface following left upper division segmentectomy. This very rare case was difficult to distinguish from thoracic abscess.

**Case presentation:**

A 68-year-old man with hypertension had undergone video-assisted thoracoscopic left upper division segmentectomy for suspected lung carcinoma. Deep vein thrombosis of the lower leg was identified and edoxaban, a so-called novel oral anticoagulant, was started on postoperative day 7. The chest drainage tube was removed on postoperative day 12 because of persistent air leakage, but fever appeared the same day. Computed tomography revealed a cavity with mixed air and fluid, so antibiotics were started on suspicion of abscess. Computed tomography-guided drainage was attempted, but proved unsuccessful. Fever continued and surgical investigation was therefore performed. Visceral subpleural hematoma was identified under the diaphragmatic surface of the left basal lung. We excised the pleura, then performed drainage and applied running sutures. The parenchyma and visceral pleura were covered with polyglycolic acid sheet and fibrin glue. Edoxaban was restarted on postoperative day 12 of video-assisted thoracoscopic surgery and no recurrence of hematoma has been revealed.

**Conclusions:**

Visceral subpleural hematoma after thoracic surgery is extremely rare. Furthermore, correct diagnosis was difficult and surgery offered a good diagnostic and therapeutic procedure.

## Background

Pulmonary visceral subpleural hematoma is rare, and has also been reported as pulmonary pseudocyst [1–4]. These lesions are caused by trauma, but postoperative visceral subpleural hematoma is extremely rare. We report a case of visceral subpleural hematoma of the left diaphragmatic surface following left upper division segmentectomy. Distinguishing pulmonary visceral subpleural hematoma from thoracic abscess is difficult. We performed video-assisted thoracoscopic surgery (VATS) to diagnose and treat our case.

## Case presentation

**Fig. 1:**
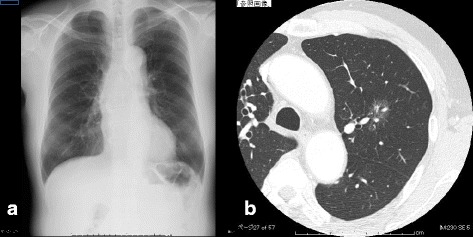
Preoperative imaging. **a** Preoperative chest X-ray reveals no abnormal sign. **b** Preoperative CT reveals a 30-mm ground glass nodule left upper lobe

The patient was a 68-year-old man with hypertension. He was not receiving anticoagulants and had no history of smoking. Chest X-ray in a regular check-up revealed an abnormal chest shadow and he was referred to our department. A 30-mm ground glass nodule in the left upper lobe (S1 + 2) was identified on chest computed tomography (CT) and lung adenocarcinoma was suspected (Fig. 1). Fluorodeoxyglucose (FDG)-positron emission tomography (PET), brain magnetic resonance imaging and transbronchial biopsy were performed, revealing left upper lung adenocarcinoma classified as cT1bN0M0 Stage IA according to Union for International Cancer Control classification (seventh edition). Preoperative laboratory data revealed no significant abnormalities of blood coagulation and thrombocytopenia. Chronic deep vein thrombosis limited to the right calf was identified on ultrasonographic screening of the veins of the lower extremities and was followed up.

**Fig. 2:**
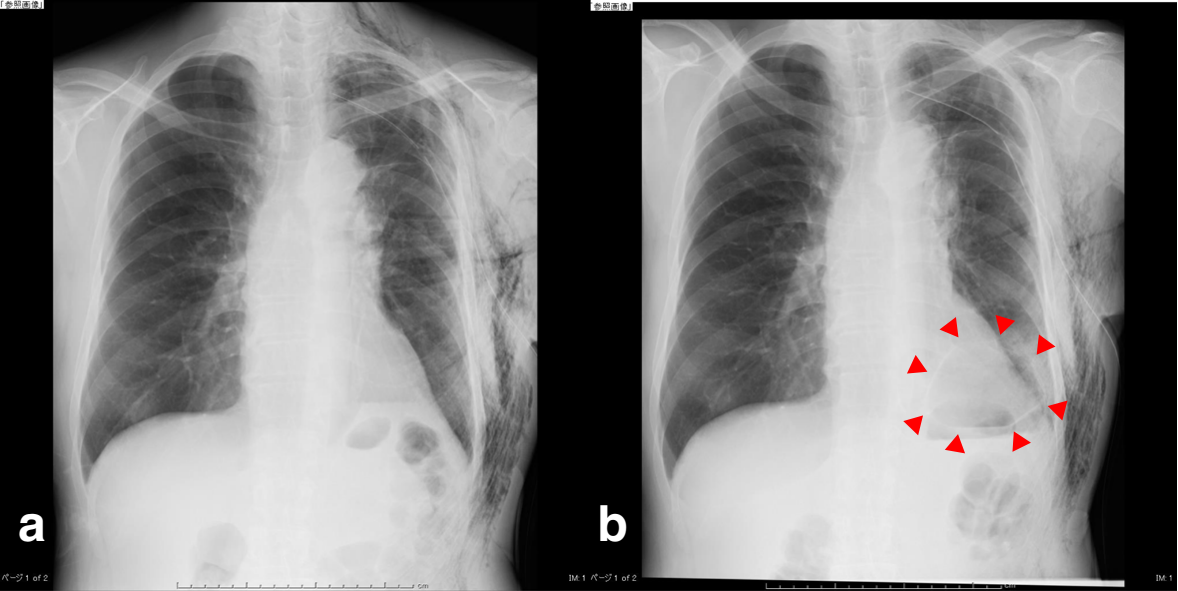
Postoperative imaging. **a** Chest X-ray on POD7 reveals no dead space in the left lung field. **b** Chest X-ray on POD12 reveals a cavity in the left lower lung field (red arrowheads)

**Fig. 3:**
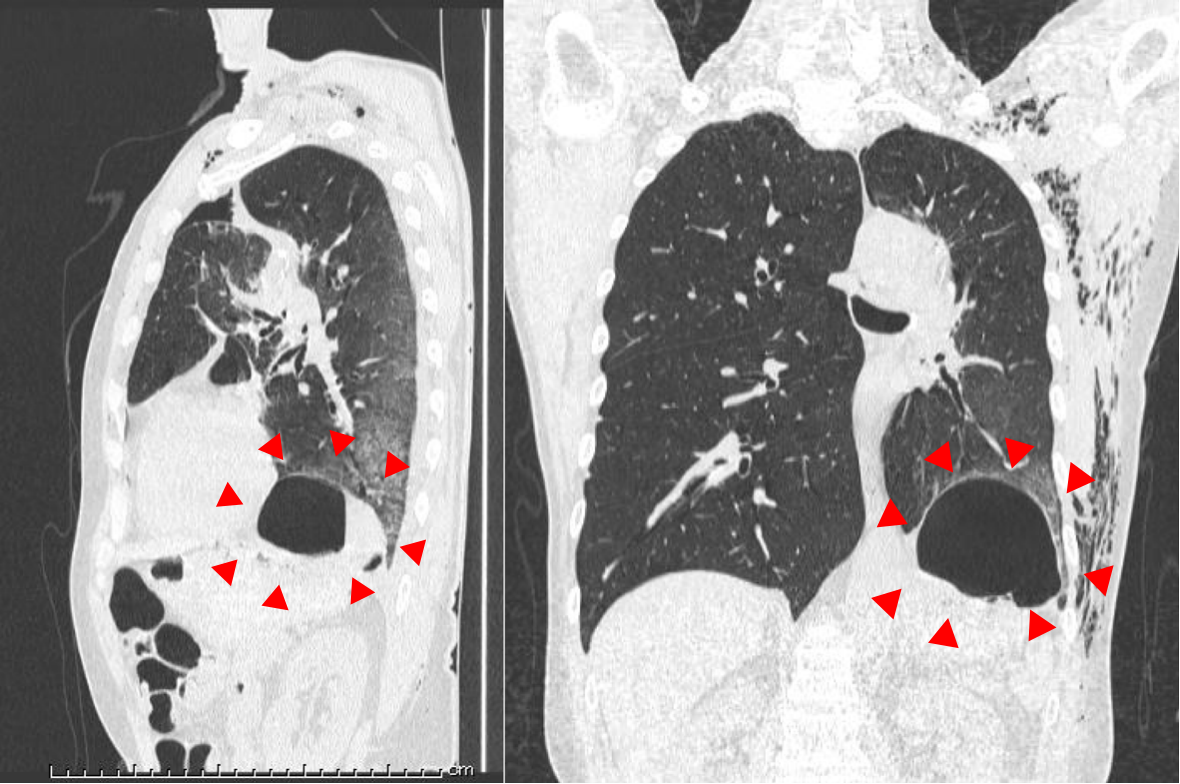
Chest CT reveals a dead space including air and fluid above the left diaphragm (red arrowheads)

**Fig. 4:**
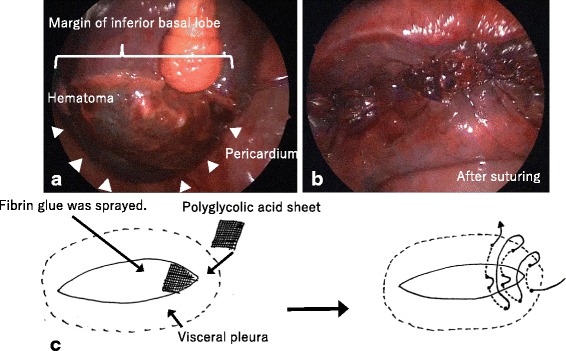
Intraoperative pictures and schema. **a** Pulmonary visceral pleural hematoma of diaphragmatic surface. **b** After suturing. **c** Procedure for the hematoma

The lesion was not a solid nodule and showed no uptake on FDG-PET. We therefore performed left upper division segmentectomy. Intraoperatively, adhesions and incomplete lobulation were not identified. Lymphadenectomy (ND2a-2) was performed. Inferior pulmonary ligament was not sectioned. Air leakage was observed on postoperative day (POD)2 and continuous low-pressure negative suction (−5 cmH_2_O) was initiated. On POD4, we changed the drainage suction water seal. Follow-up ultrasonography of the lower extremity revealed new calf-limited deep vein thrombosis on POD7 and the novel oral anticoagulant edoxaban was started. The thoracic drain was clamped on POD10 and removed on POD12. Fever of 38°C appeared the same day, but no other symptoms were identified. A cavity appeared in the left lower lung field on chest X-ray (Fig. 2). Chest CT revealed a dead space including air and fluid above the left diaphragm and edoxaban was stopped (Fig. 3). CT-guided pleurocentesis was performed on POD13, but the space collapsed and proved impossible to drain. As fever persisted, thoracoscopic surgery was performed. No adhesions were seen on the left lower lobe, but slight pleural effusion and visceral subpleural hematoma of the basal lung were identified. The basal pleura was excised with an electrosurgical knife and the hematoma was removed. The air leak point was sutured, then a polyglycolic acid sheet was fixed to the lung parenchyma and sprayed with fibrin glue. The pleura and parenchyma were then sutured with running sutures (Fig. 4).

**Fig. 5:**
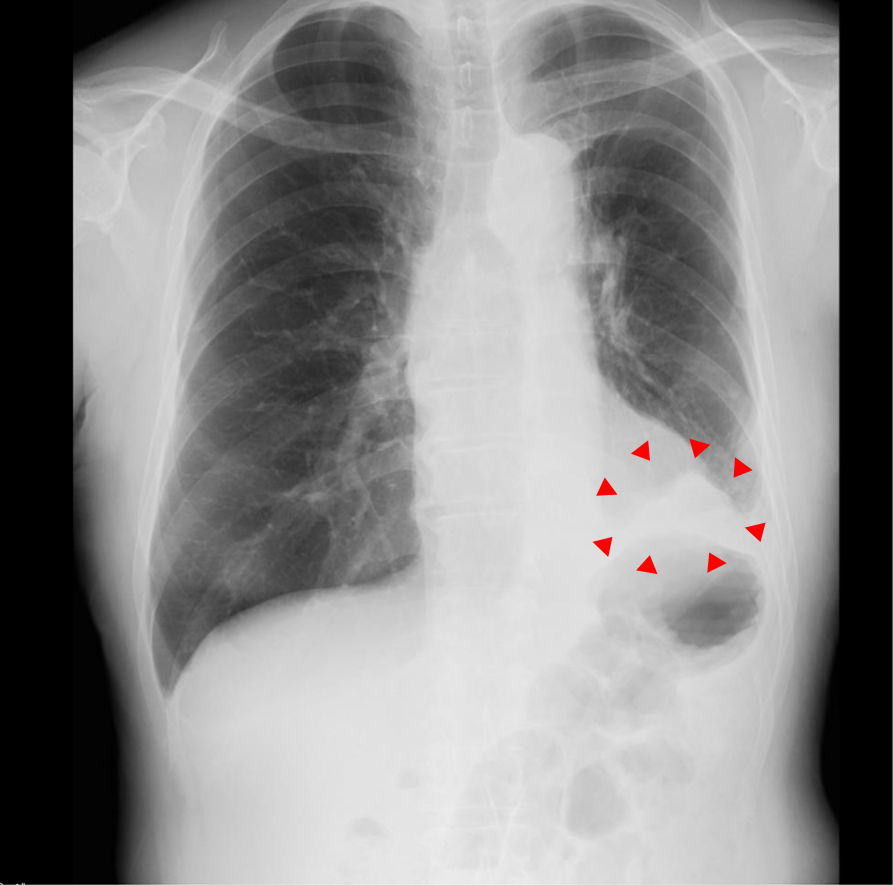
Chest X-ray after two months edoxaban was restarted

On POD2 after the second operation, the drainage tube was removed. The patient was discharged on POD8. Cultures of fluid and pleural effusion yielded negative results. Edoxaban was restarted on POD12, but no recurrence of hematoma has been found over one year of follow-up in the outpatient clinic (Fig. 5).

## Discussion

We reported a very rare case of postoperative visceral subpleural hematoma. Diagnosis was difficult and surgery was required. Subpleural hematoma is classified as either parietal subpleural hematoma or visceral subpleural hematoma. Hematomas of the parietal pleura account for most reports of subpleural hematoma. Visceral pleural hematoma was reported in three of 98 patients who underwent preoperative CT-guided hook wire localization [5]. Visceral pleural hematoma was seen in 1 case among 138 pleural complications in lung transplant recipients [6]. Traumatic false aneurysm of the descending aorta that ruptured into the right thoracic cavity has also been reported to result in visceral pleural hematoma [7]. Given these findings, visceral subpleural hematoma is rare and has also been reported as pulmonary pseudocyst or cavitary pulmonary lesions [1]. Most cases were reported as traumatic pulmonary pseudocyst and contained fluid and air [1–3]. Such lesions are caused by laceration of the visceral pleura in lung injuries after blunt chest trauma, and are most often seen in children or young adults. Because the thorax is elastic, the visceral pleura remains intact, and the parenchyma is easily injured [2]. Although these cases have been reported after trauma, those following lung resection are very rare. Hematoma in the intersegmental plane has been reported after segmentectomy, where the surgeon attached the borders of the intersegmental plane to each other [8]. To the best of our knowledge, no cases of postoperative subpleural hematoma have been described in the English literature. A case of visceral subpleural hematoma after lobectomy for rapidly growing metastatic lung tumor has been reported in Japanese [9]. Visceral subpleural hematoma in this case was revealed on the interlobar surface near the staple line. However, visceral subpleural hematoma in our case appeared in the subpleura of the basal lung, and was not touched in the first operation. In our case, edoxaban was started and a dead space with an air-fluid level subsequently appeared on chest X-ray. We considered that the small hematoma appeared under anticoagulation due to pressure changes under diaphragmatic movements such as cough, and progression of laceration, trapping air and increasing in size. On the other hand, pulmonary visceral hematoma may have arisen from pulmonary pseudocyst, in which case the cause cannot be identified.

The chest X-ray appearance of visceral subpleural hematoma sometimes resembles that of lung abscess, cavitating tuberculosis, or bronchial carcinoma with cavitation in adults [3]. In particular, computed tomography may be helpful in diagnosing traumatic pulmonary pseudocyst [1]. Treatment usually involves conservative therapy, as the cyst contents are excreted or absorbed spontaneously. Surgery is required if the pseudocyst develops persistent infection [2]. Our case could not be differentiated from abscess. Moreover CT-guided drainage was also difficult. Because we thought that the point of puncture was limited and the insertion of a puncture needle was difficult on account of the cyst with elastic feature. Surgery proved useful as a diagnostic and therapeutic procedure. There may be the other opinion such as the surgical procedure which covers the diaphragmatic surface of the lower lobe with tissue adhesives without running suture after removing the peel of the visceral pleura. However, we thought that a running suture could prevent the visceral pleura peeling off more, and we selected the procedure.

## Conclusions

Postoperative visceral subpleural hematoma distant from the operative site is extremely rare. Furthermore, correct diagnosis was difficult. Surgery is a good diagnostic and therapeutic procedure.

## Abbreviations

CT: computed tomography
FDG-PET: fluorodeoxyglucose-position emission tomography
VATS: video-assisted thoracoscopic surgery
